# Electrochemical analysis of anionic analytes in weakly supported media using electron transfer promotion effect: a case study on nitrite

**DOI:** 10.1038/s41598-020-71365-4

**Published:** 2020-09-03

**Authors:** Alireza Khoshroo, Ali Fattahi

**Affiliations:** 1 Pharmaceutical Sciences Research Center, Health Institute, Kermanshah University of Medical Sciences, Bākhtarān, Iran; 2 Medical Biology Research Center, Health Technology Institute, Kermanshah University of Medical Sciences, Bākhtarān, Iran

**Keywords:** Environmental sciences, Chemistry

## Abstract

In this study, a simple technique was developed for the electrochemical detection of anionic analytes in weakly supported media. This was conducted by the use of electrochemical paper-based analytical devices (ePADs). A sensing platform was modified with nereistoxin and used to determine nitrite as a case study. The electrochemical response was improved due to the accelerated electron transfer between the sensing platform and the nitrite through the electrostatic interaction of the amino group of nereistoxin and the nitrite. The electrocatalytic current of the nitrite in the presence of nereistoxin was enhanced in the weakly supported media. By using nereistoxin as a signal enhancer, 97% of the electrochemical signal was obtained at the low ionic strength of the electrolyte, while less than 35% of this signal was obtained in the absence of nereistoxin. The limit of detection was as low as 20 nM using an ePAD. Generally, the proposed ePAD serves as a promising, efficient and low-cost device for sensing applications in weakly supported media.

## Introduction

The development of analytical methods for the detection of various analytes is essential in environmental analysis and clinical research^1–3^. Any novel method for this purpose needs to be sensitive, accurate, easy to practice and inexpensive^4–7^. Among various analytical methods, the use of electrochemical paper-based analytical devices (ePAD) has attracted significant attention. This is due to the unique characteristics of those devices such as simple fabrication processes as well as economical and compact systems^8–10^. So far, various methods have been used to fabricate paper-based systems such as inkjet printing^11^, photolithography^12^, wax printing^13^, PDMS plotting^14^, plasma etching^15^ and laser treatment^16^. Most of these methods require harmful chemical materials and complicated fabrication processes to create hydrophobic areas on the paper^17,18^. Hence, a simple and rapid prototyping technique is essential for the construction of ePAD, specifically in practical applications. Besides, providing user-friendly and low-cost conductive inks is a crucial component in the process of constructing ePADs. To date, many materials, e.g., graphite^19^, graphene^20^, carbon nanotube (CNT)^21^ and silver nanoparticles^22^, have been widely used to prepare conductive inks. Among these materials, carbon-based electrodes prove to have low background currents and broad electrochemical windows.

In addition to a proper method of fabricating ePADs, a new detection procedure should be introduced to enhance the sensitivity of ePAD or facilitate sample preparation. Despite the significance of developing new electrochemical sensors for various analysts, most papers in the field have just reported the application of available electrochemical sensors in weakly supported media^23,24^. Compton et al.^23^ showed that the kinetics of electrochemical reactions might be affected by the concentration of the supporting media. The electric field on the electrode surface decreased with the decrease of the supporting medium concentration, which led to a change in the peak current and the ΔEp^25^. In another study^24^, a laccase-based microscale sensor was designed to determine phenol in weakly supported media. The advantage of this microsystem was the formation of convergent diffusion on the surface of microelectrodes rather than the planar mass transfer on macroscale electrodes.

The present study aims at the development of an electrochemical paper-based analytical device (ePAD) for the detection of anionic analytes in weakly supported media. Although nitrite is widely used for various applications, it is considered toxic by the World Health Organization (WHO), and its maximum recommended concentration in drinking water should be 13 mg L^−1^^26^. Therefore, it is crucial to develop an analytical method for nitrite monitoring in real samples. To serve the purpose, this paper reports the application of a straightforward, low-cost, and highly sensitive ePAD for the reliable detection of nitrite. The ePAD is modified with a nereistoxin and works through the electrostatic interaction between the amino group of nereistoxin and nitrite. The catalytic signals are highly improved in the presence of nereistoxin, leading to an accelerated electron transfer. The fabricated ePAD has proved to be applicable for the detection and analysis of nitrite in weakly supported media.

## Results and discussion

### Fabrication of the ePAD

An ePAD was fabricated by the sticking of a patterned self-adhesive glossy paper onto the surface of a commercial type of wax paper. The different steps of the fabrication process are shown in Fig. S1 (Supporting Information). The patterned self-adhesive glossy paper was used as a stencil to control the size and the shape of the electrode system. Furthermore, this layer could provide a hydrophobic area on the ePAD. Basically, the reproducibility of the surface area of an electrode is a critical parameter for the development of an electrochemical sensor. The laser cutting technique provides a precise stencil from glossy adhesive paper. The reproducibility of the surface area of electrodes also depends on laser cutter parameters, including power and speed. In this study, the effects of these parameters on the reproducibility of the patterns were evaluated by the ImageJ software. The mean calculated for the surface area in ten patterns and the relative standard deviations (RSD) are listed in Tables S1 and S2. The RSD values calculated for the optimum parameters (i.e. the power of 12.5% and the speed of 10 mm/s) were around 4%. Therefore, this method can be applied in the reproducible construction of ePADs.

**Figure 1:**
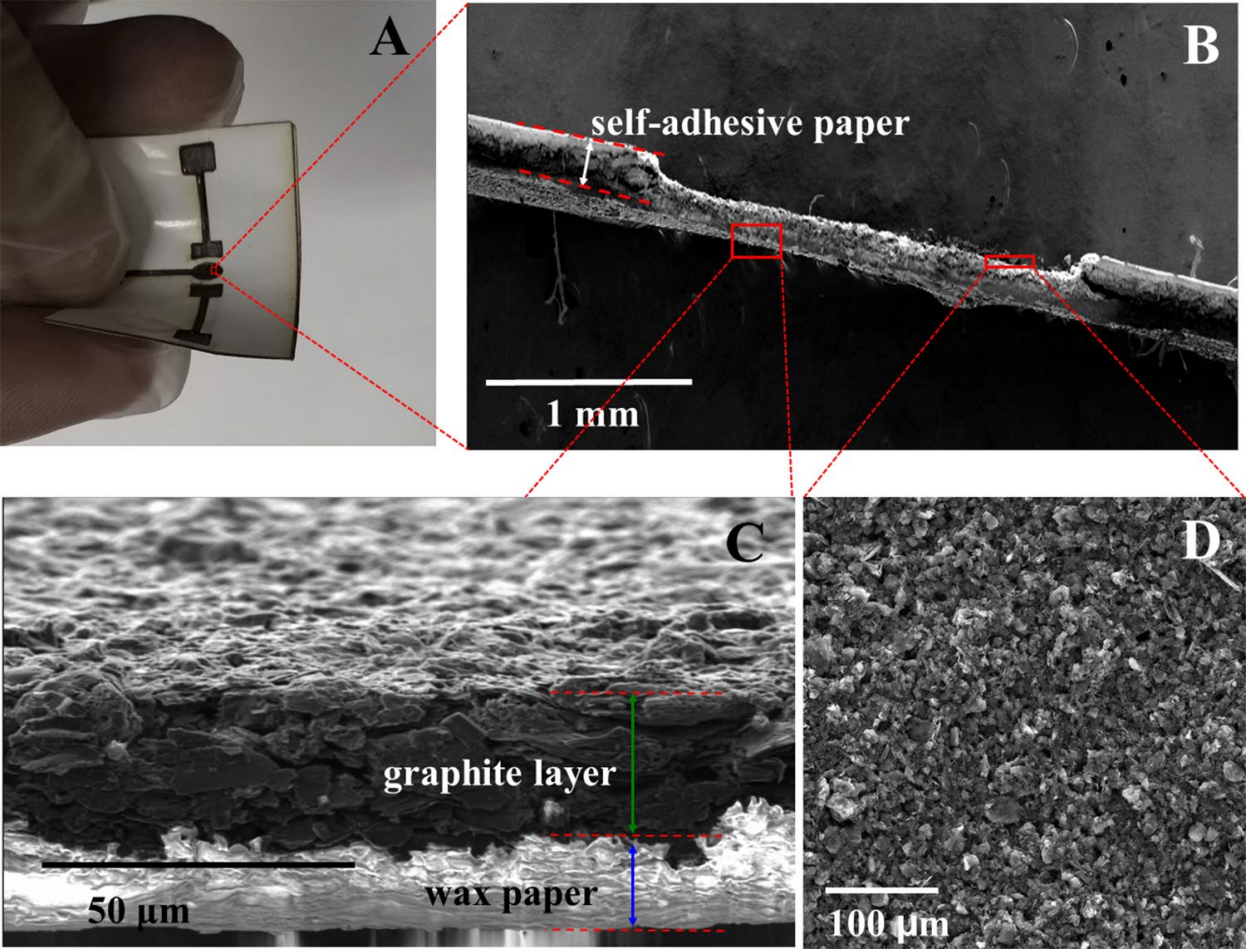
(**A**) ePAD with the flexible paper, SEM images of cross-section (**B**,**C**) and top view (**D**) of ePAD.

In the next step (Fig. S1e,f), the electrodes and the connections were fabricated by a manual screen-printing process. The as-fabricated ePAD was mechanically stable and flexible (Fig. 1A). The thickness of the layer of graphite was investigated using SEM (Fig. 1B–D). It could be controlled easily by adjusting the pattern thickness. The SEM image showed the uniform printing of the graphite layer on the paper surface with a thickness of 40.5 ± 1.9 μm. The reproducible paper-based electrochemical system could, thus, be fabricated using the proposed method.

The surface morphology of the working electrode before and after the deposition of AuNPs was observed by SEM (Fig. S2). The AuNPs were successfully distributed onto the graphite surface with a sphere-like structure. Figure S3a shows the X-ray diffraction spectra of the Au/ePAD. The diffraction peaks located at 2θ = 43.2° correspond to (100) crystal planes of graphite, while the diffraction peaks at around 2θ = 36.3°, 38.1°, 50.4°, and 74.1° can be assigned to (111), (200), (220) and (311) planes of the Au nanoparticles respectively^27,28^. These results suggest the formation of Au nanoparticles on the graphite working electrode surface. Furthermore, as the energy-dispersive X-ray spectrum of the Au/ePAD suggests in Fig. S3b, the working electrode surface was composed of Au and carbon.

### Electrochemical investigation of the ePAD

**Figure 2:**
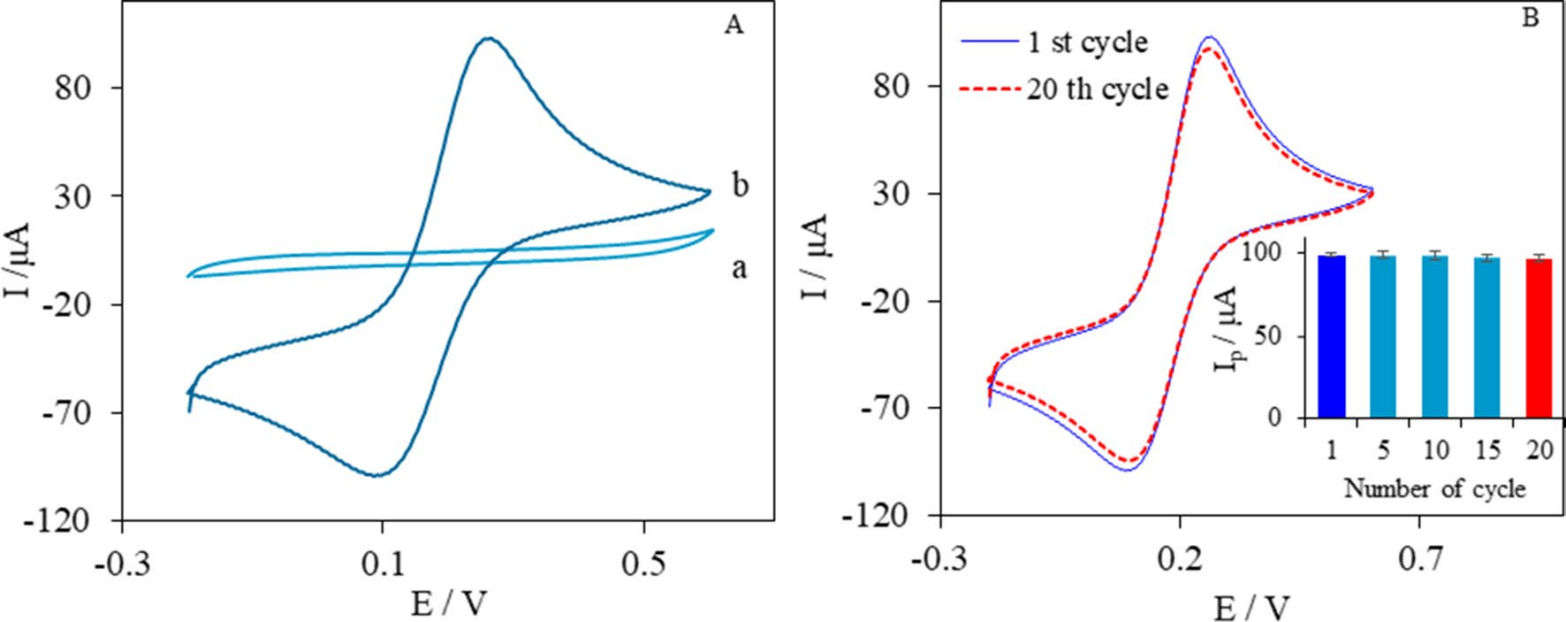
(**A**) CVs of ePAD in the absence (a) and presence (b) of 1.0 mM Fe(CN)^3−/4−^ containing 0.1 M KCl at a scan rate of 100 mV s^−1^. (**B**) 1st cycle and the 20th cycle of ePAD in 1.0 mM Fe(CN)^3−/4−^ containing 0.1 M KCl at a scan rate of 100 mV s^−1^.

Cyclic voltammetry was conducted to investigate the electrochemical performance of the ePAD in the presence of 1.0 mM Fe(CN)^3−/4−^ containing 0.1 M KCl at a scan rate of 100 mV s^−1^*.* The cyclic voltammogram in the absence of electroactive species (curve a, Fig. 2A) showed a low background current without an oxidation or reduction peak. This indicates that graphite ink components are not electroactive in the CVs potential range. However, in the presence of 1.0 mM [Fe(CN)_6_]^4−/3−^, a pair of redox peaks appeared with the ΔE_p_ of 176 mV (curve b in Fig. 2A). This suggests that [Fe(CN)_6_]^4−/3−^ has a quasi-reversible behavior. In the literature, ΔE_p_ values higher than 150 mV have been reported for graphite-based electrodes^19,29,30^, which is attributed to the high electrical resistance of the binder^31^. Furthermore, the ratios of I_pa_ to I_pc_ for the 1st and the 20th cycles were 1.05 and 1.02, indicating the stability of ferricyanide ions produced at the surface of the electrode. It is noteworthy that no absorption or adsorption of [Fe(CN)_6_]^4−/3−^ ions occurred on the graphite working electrode of the ePAD.

Electrochemical stability is an important parameter for the development of various electrochemical sensors, especially paper-based sensors. In this case, after 20 cycles on the ePAD, the peak potentials did not have a noticeable change, and the peak currents only varied for less than 2% (Fig. 2B). These results suggest that ePADs have acceptable electrochemical stability to be applied as sensors.

The day-to-day reproducibility of inks was investigated by the measurement of the peak currents for the ePADs constructed on different days (Fig. S4). The RSD values calculated for different inks were around 5%, indicating that the process of ink preparation had good reproducibility. Moreover, the RSDs of five replicates from a single-batch ink were less than 3%.

**Figure 3:**
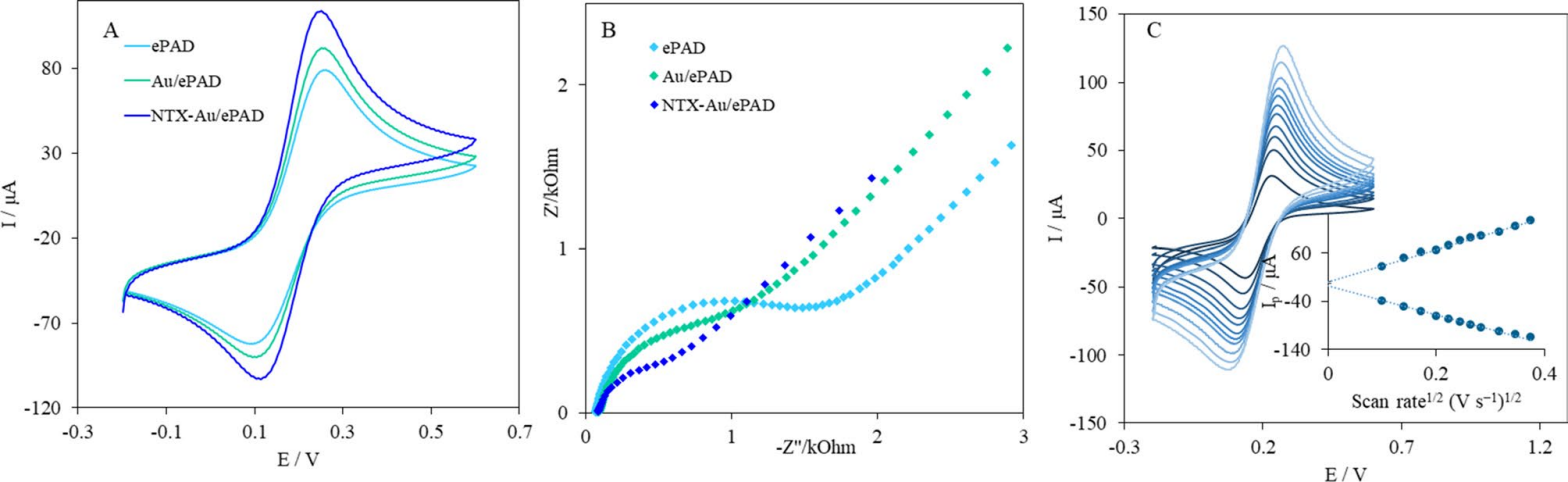
(**A**) CVs ePAD, Au/ePAD and NTX–Au/ePAD in 1.0 mM Fe(CN)^3−/4−^ containing 0.1 M KCl at a scan rate of 100 mV s^−1^, and (**B**) EIS of ePAD, Au/ePAD and NTX–Au/ePAD in 1.0 mM Fe(CN)^3−/4−^ containing 0.1 M KCl, (**C**) CVs of NTX–Au/ePAD in 1.0 mM Fe(CN)^3−/4−^ containing 0.1 M KCl at different scan rates, from 10 to 140 mV s^−1^, Inset: Variation of Ip vs. logarithm of ν^1/2^.

The electrochemical characterization of ePAD, Au/ePAD, and NTX–Au/ePAD was performed by cyclic voltammetry in the presence of 1.0 mM [Fe(CN)_6_]^4−/3−^ containing 0.1 M KCl at a scan rate of 100 mV s^−1^. A pair of well-defined redox peaks was observed at the ePAD (Fig. 3A). When the Au nanoparticles were deposited on the graphite working electrode, the electroactive surface was increased, and the peak current was enhanced consequently. After the modification of AuNPs with NTX (NTX–Au/ePAD), due to the acceleration of the electron transfer between [Fe(CN)_6_]^4−/3−^ in solution and the electrode surface, the peak current improved significantly; the value of ΔE_p_ for the NTX–Au/ePAD (ΔE_p_ = 149 mV) was smaller than that for the Au/ePAD (ΔE_p_ = 160 mV) and the ePAD (ΔE_p_ = 176 mV).

The interface properties of NTX–AuNPs on the ePAD surface were evaluated using electrochemical impedance spectroscopy (EIS). Figure 3B shows the Nyquist plots for the ePAD, the Au/ePAD and the NTX–Au/ePAD. The R_ct_ was decreased as AuNPs and NTX were added to the ePAD and the Au/ePAD respectively, suggesting that the presence of NTX on AuNPs effectively enhanced the electron transfer of the electrode. These results confirm the assembly of NTX and AuNPs on the surface of the working electrode.

In order to understand the nature of the redox currents, the cyclic voltammograms of the NTX–Au/ePAD were recorded at various scan rates. Figure 3C shows the cyclic voltammograms of the NTX–Au/ePAD in the presence of 1.0 mM Fe(CN)^3−/4−^ containing 0.1 M KCl at different scan rates (ν). The figure also denotes the dependence of cathodic and anodic peak currents on the square root of scan rates. The linearity of the cathodic and anodic peak currents vs. the ν^1/2^ suggests that electrochemical reactions on NTX–Au/ePADs are diffusion-controlled rather than adsorption-controlled.

### Electrochemical behaviors of nitrite at the NTX–Au/ePAD

**Figure 4:**
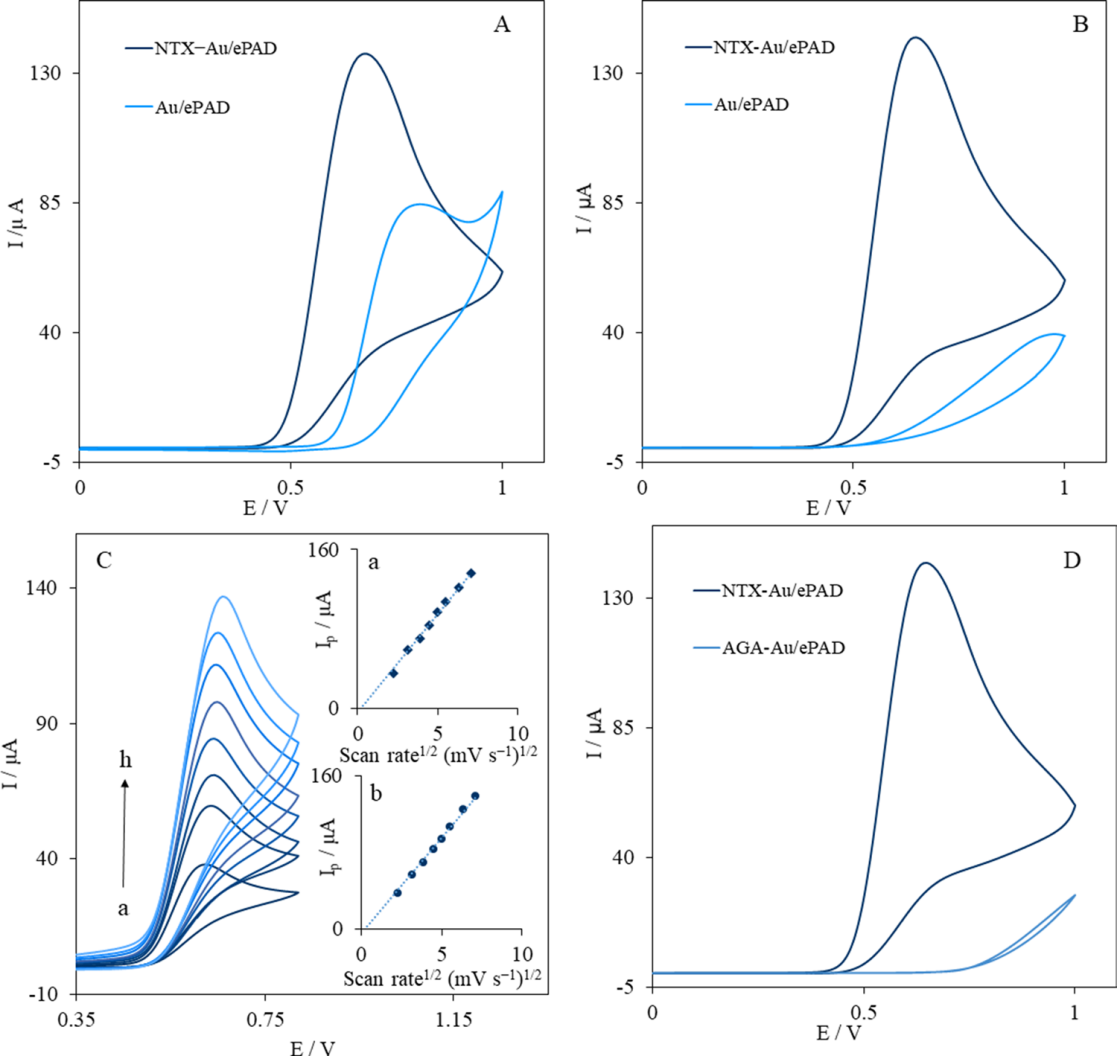
CVs of Au/ePAD and NTX–Au/ePAD in 0.1 M (**A**) and 0.001 M (**B**) phosphate buffer solution (pH 6.0) in the presence of 1.0 mM nitrite at a scan rate of 50 mV s^−1^. (**C**) CVs of NTX–Au/ePAD at different scan rates, from 5 to 50 mV s^−1^ in 0.1 M phosphate buffer solution (pH 6.0) in the presence of 1.0 mM nitrite, Inset: Variation of Ip vs. logarithm of ν^1/2^. (**D**) CVs of NTX–Au/ePAD and AGA–Au/ePAD in 0.001 M phosphate buffer solution (pH 6.0) in the presence of 1.0 mM nitrite.

The electrochemical activity of ePADs for the oxidation of nitrite was investigated with CVs in the presence of various concentrations of a phosphate buffer solution (pH 6.0) in the potential range between 0.0 to 1.0 V at a scan rate of 50 mV s^−1^ (Fig. 4A,B). The Au/ePAD presented an oxidation peak for 1.0 mM nitrite in a 0.1 M phosphate buffer solution (pH 6.0) at 805 mV. After the modification of AuNPs with NTX, the oxidation peak current increased obviously (I_p_ had a 61% improvement) with a 120-mV negative shift at the peak potential (Fig. 4A). The significant improvement in the nitrite oxidation signals indicated that NTX–AuNPs could effectively enhance the electrochemical performance of the ePAD.

The oxidation of nitrite in a low concentration of the supporting electrolyte (0.001 M phosphate buffer, pH 6.0) was investigated (Fig. 4B). The oxidation current of 0.1 mM nitrite at the Au/ePAD sharply decreased as the supporting electrolyte concentration decreased at 0.001 M. Nevertheless, in the presence of NTX, the oxidation current did not change with the concentration of the supporting electrolyte (Fig. 4B). To investigate the transfer of nitrite ions to the electrode surface, the oxidation of 1.0 mM nitrite was recorded at various scan rates. Figure 4C shows the cyclic voltammograms of the NTX–Au/ePAD in the presence of 1.0 mM nitrite ions in a 0.1 M phosphate buffer solution (pH 6.0) at different scan rates. The linearity of the oxidation currents vs. the scan-rate^1/2^ suggested that the electrochemical oxidation of nitrite was controlled by diffusion from the solution to the surface of the working electrode (Inset a of Fig. 4C). Also, a linear correlation between currents vs. the scan-rate^1/2^ was observed in the 0.001 M PBS, which indicated that the oxidation of nitrite was controlled by diffusion from the solution to the surface of electrode (Inset b of Fig. 4C). Furthermore, the diffusion coefficient (D) for the nitrite ions at the NTX–Au/ePAD was calculated by the Randles–Sevcik equation^32^. The value of the diffusion coefficient for nitrite ions on the NTX–Au/ePAD was 1.44 × 10^−5^ cm^2^ s^−1^.

**Figure 5:**
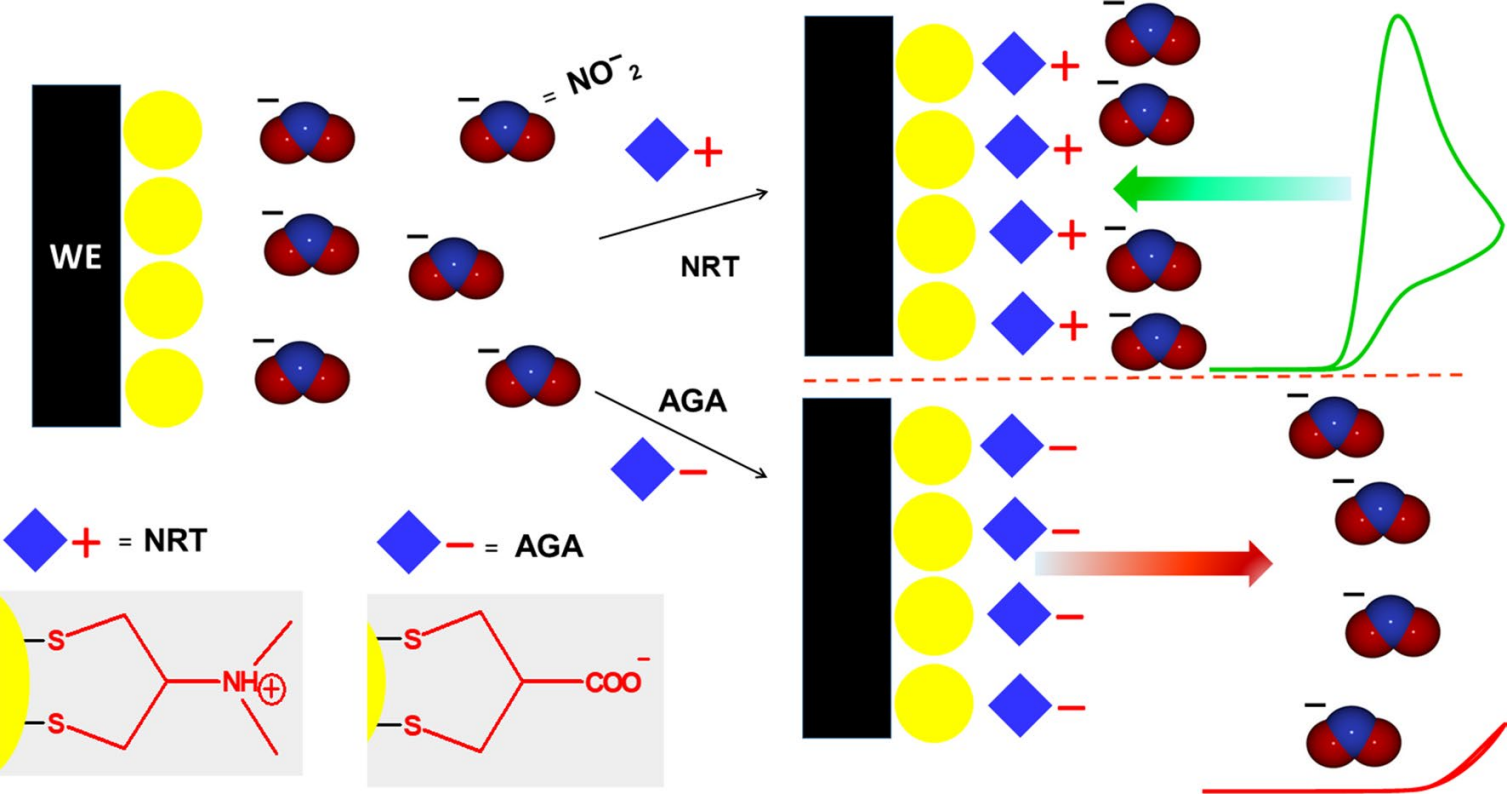
The mechanisms of nitrite reaction at NTX–Au/ePAD and AGA–Au/ePAD.

Based on the results, the presence of NTX on AuNPs leads to the acceleration of electron transfer kinetics with a low ionic strength. Under weakly supported conditions, the migration of ions leads to electroneutrality with changes in the concentration profiles of the ions on the electrode surface. In the typical concentration of the supporting electrolyte, the ions migrate in the electrolyte and perturb the electric field. Therefore, analytes with a low concentration can diffuse on the electric double layer^23^. While the electron transfer of nitrite ions in the absence of NTX decreases by this effect, the positively charged amino group on the NTX provides a potential gradient on the electrode surface, similar to the one obtained in a typical concentration of the supporting electrolyte^33^. Furthermore, through an electrostatic interaction, this positively charged NTX leads to the concentration of nitrite ions near the surface, as shown in Fig. 5.

In order to confirm this effect, the working electrode of the ePAD was modified with asparagusic acid (AGA) instead of NTX in the same experimental conditions (Fig. 4D). Asparagusic acid is a derivative of disulfide compounds with a carboxylic acid functional group. As it can be seen in Fig. 4D, there is no peak current for the oxidation of 1.0 mM nitrite with AGA–AuNPs. The peak current disappeared with the adsorption of AGA on AuNPs, indicating that the access of nitrite to the electrode surface was suppressed by AGA. This may be attributed to the electrostatic repulsion of the carboxylate group of AGA on the AuNPs surface (Fig. 5).

### Optimization of the experimental conditions

The effects of the experimental conditions including pH, incubation time and concentration of NTX on the performance of the NTX–Au/ePAD were investigated. The corresponding details are presented in Fig. S5. The effect of pH on the electrocatalytic oxidation of 1.0 mM nitrite was investigated using the NTX–Au/ePAD at different pH values between 2 and 9. Based on the results, with an increase in the pH from 2.0 to 6.0, the peak currents increased and then decreased (Fig. S5). The maximum peak current occurred at pH 6. The decrease in the oxidation peaks at pH values lower than 6 was due to the decomposition of nitrite ions to NO^3–^, as reported in previous papers^34,35^. Besides, the sharp decrease in the currents at a pH value higher than 6 was due to the lack of protons^36^.

The effect of the incubation time of NTX on the oxidation of 1.0 mM nitrite was also investigated, and the optimum time of 7 min was obtained. The impact of pH on the NTX absorbed on the AuNPs was also evaluated. The oxidation peak currents of nitrite increased with an increase in the pH of NTX solutions. This may be due to the dependence of NTX ionization on pH, which leads to increased hydrophobicity of NTX at higher pH values, as well as the accelerating effect of the NTX adsorbed on the AuNPs. Therefore, pH 9 was used for the NTX solutions.

### Calibration curve of nitrite

**Figure 6:**
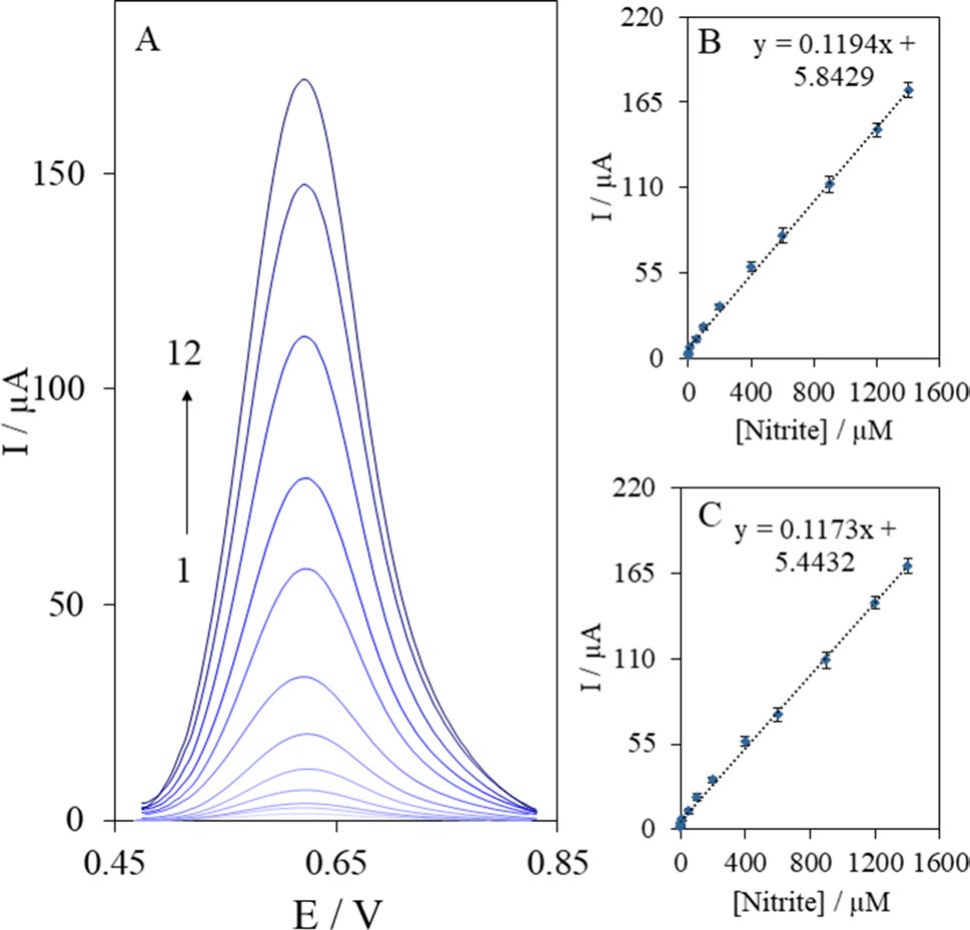
(**A**) DPVs of NTX–Au/ePAD in 0.1 M phosphate buffer solution (pH 6.0) in different concentrations of nitrite, numbers 1–12 correspond to 0.05–1,400 µM, Plot of the peak currents as a function of nitrite concentration in 0.1 M (**B**) and 0.001 M (**C**) phosphate buffer solution (pH 6.0). DPV parameters: amplitude, 0.05 V; pulse width, 0.2 s; sampling width, 0.05 s; pulse period, 0.5 s.

Figure 6A shows the DPV curves of the NTX–Au/ePAD in different concentrations of nitrite in a 0.1 M phosphate buffer solution (pH 6.0). The peak currents of the nitrite oxidation increased with an increase in the nitrite concentration. Besides, the calibration plot of the nitrite concentration versus the oxidation peak currents presented in Fig. 6B indicates a linear relationship between the peak currents and the nitrite concentration in a range from 0.05 to 1,400 μM with the linear regression equation of I_p_ (μA) = 0.1194 C_nitrite_ (μM) + 5.84. The limit of detection calculated from the calibration plot (3s_b_/slope) was found to be 20 ± 2 nM. The calibration plot in the weakly supported media (0.001 M PBS) was obtained too (Fig. 6C). The regression equation was I_p_(μA) = 0.1173 C_nitrite_(μM) + 5.44. The slopes of the calibration curves for nitrite in the normally supported media (0.1119 μA μM^−1^) and the weakly supported media (0.1173 μA μM^−1^) were virtually the same, which indicates that the determination of nitrite is possible in weakly supported media too. Table S3 summarizes the analytical performance of the proposed method based on an NTX–Au/ePAD with some electrochemical sensors for nitrite. The proposed NTX–Au/ePAD provides a sensitive technique for the determination of nitrite in real samples.

### The selectivity, repeatability, stability and analytical applications of NTX–Au/ePADs

The selectivity of the NTX–Au/ePAD was performed in the presence of various interfering chemicals that may co-exist with nitrite in samples. A 20-fold concentration of NaCl, Na_2_CO_3_, MgCl_2_, CuSO_4_, FeCl_2_, FeCl_3_, Na_2_HPO_4_, CH_3_COONa and Na_2_CO_3_ and a tenfold concentration of cysteine, ethanol, methanol, uric acid, ascorbic acid, glucose and urea were added to a mixture of 0.1 mM nitrite. Then, the NTX–Au/ePAD was used for measurements. Based on the results, no relative signal change over ± 5% could be observed, which suggests that the NTX–Au/ePAD was not affected by the interfering chemicals that co-existed with nitrite in the samples.

The reproducibility of the method for the oxidation of 50 μM nitrite was investigated using twelve NTX–Au/ePADs. The relative standard deviation (RSD) was 4.6%, indicating the acceptable reproducibility of the NTX–Au/ePAD. Moreover, the RSD for three determinations of 50 µM nitrite using a single NTX–Au/ePAD was 2.6%, indicating the good repeatability of the device. In fact, a laser cutter provides a precise and rapid prototyped pattern which makes it easy to print the ink on the wax paper and to control the thickness and the surface area of the printed ink.

Furthermore, the NTX-Au/ePAD was used to measure nitrite in real samples. As presented in Table S4, the response obtained from the NTX-Au/ePAD was comparable with those obtained by the Griess protocol. T-tests indicated no significant differences between the NTX-Au/ePAD and the Griess method at a confidence level of 95%.

## Conclusion

In this study, a low-cost method was developed to construct a paper-based analytical device using a patterned sticker on wax paper. The sensing platform of an ePAD was modified with nereistoxin, and then the device was used to determine nitrite in weakly supported media. As it was found, the positively charged amino group on NTX provides a potential gradient on the working electrode, similar to the one obtained in a normal concentration of a supporting electrolyte. Through an electrostatic interaction, the positive charge of NTX leads to the concentration of nitrite ions near the surface. Using this finding, it is possible to introduce an electrochemical platform for the detection of anionic analytes in weakly supported media.

## Experimental

### Chemicals and apparatus

Silver nitrate, graphite powder, potassium chloride, potassium ferrocyanide, acetone, pro-analysis grade phosphate salt, sodium hydroxide, and the required reagents were obtained from Merck (Darmstadt, Germany). Colorless nail polish was purchased from Arika, French. A4 self-adhesive glossy paper was supplied from Taha label, Iran, and a laser cutter (Mini Laser BCL-MU, Jinan Bodor CNC Machine Co, China) was used to prepare stickers. Wax paper was obtained from Iran Package Co. (Tehran, Iran). The morphology of the surface was investigated by scanning electron microscopy (TESCAN, Czech Republic). An EDX (BRUKER) was used to analyze the elemental composition of the ePAD. The XRD spectrum of the ePAD was recorded by an APD 2000-Italian Structures X-ray generator. To carry out XRD, the samples were coated on an XRD grid. A voltage of 40 kV and a current of 30 mA were used along with Cu K^−1^ radiation. The surface area of the patterns was estimated by the ImageJ software (version 1.52a; National Institutes of Health, USA)^37^.

### Electrochemical measurements

Electrochemical studies were conducted with an Autolab PGSTAT101 potentiostat/galvanostat (Eco Chemie, Netherlands). The electrochemical analyses were performed with a three-electrode system consisting of gold NPs on a graphite layer as a working electrode, an Ag quasi-reference electrode as a reference electrode, and a graphite electrode as a counter electrode. Impedance spectra were recorded in the frequency range of 10 to 10^5^ Hz and with the amplitude of 10 mV. DPV was performed with an amplitude of 0.05 V, a pulse width of 0.2 s, a sampling width of 0.05 s, and a pulse period of 0.5 s in the range of 0.5–0.08 V.

### Preparation of electrochemical paper-based analytical devices

To fabricate an ePAD, a carbon ink had to be prepared. For this purpose, 0.7 g of graphite powder and 0.3 g of colorless nail polish were mixed up. Then, 0.5 mL of acetone was added to the mixture. They were mixed for 5 min until a homogeneous ink was obtained. The ePAD was fabricated on a commercial type of wax paper by the use of a three-electrode system consisting of a working electrode, a reference electrode and a counter electrode. The connections and the electrodes of ePAD were constructed with the lab-made carbon ink. Firstly, the electrodes and their connections were patterned on an A4 self-adhesive glossy sheet of paper using a laser cutter. For the manual screen-printing process, the patterns were stuck on a wax paper substrate, followed by the manual screen printing of the carbon ink into the patterns to provide the three electrodes and the contact pads. A squeegee was used to spread the carbon ink on the define area with the same pressure. Then, the prepared ePAD was dried in an oven for 30 min. After that, a layer of colorless nail polish, as an insulating layer, was screen printed over the connection to expose a defined area. The ePAD was kept there to dry up for an hour. The working electrode modified with gold NPs and silver ink was used to construct a quasi-reference electrode. The formation of gold nanoparticles (AuNPs) on the graphite working electrode was studied by cyclic voltammetry in the potential range of − 0.2 to + 1.5 V in a 0.5 mM HAuCl_4_ solution containing 0.5 M sulfuric acid at the scan rate of 50 mVs^−1^. The ePAD was then rinsed with water to remove the free ions from its surface. In the next step, 15 μL of a 10 μM nereistoxin (NTX) solution was placed on the working electrode of the ePAD for an optimized time at room temperature. Finally, the electrodes were washed with a 0.1 M NaOH aqueous solution to remove the species that were not physically adsorbed.

The reference electrode was constructed with Ag ink applied on the prepared graphite layer. The Ag ink was synthesized according to the method previously reported by Lewis^38^, which is based on poly(acrylic acid) and diethanolamine. The Ag quasi-reference electrode was prepared by the manual screen printing of the Ag ink. As a final step of the device fabrication, the ePAD was left to dry for an hour. The prepared device with a three-electrode system was used for further analysis.

## Supplementary information

Supplementary Information
