# Supplementary material for: Electrochemical analysis of anionic analytes in weakly supported media using electron transfer promotion effect: a case study on nitrite
Source: Sci Rep. 2020 Sep 3;10:14511. doi: 10.1038/s41598-020-71365-4 (PMC7471947; doi:10.1038/s41598-020-71365-4)
Supplement: Supplementary file 1 — Supplementary Information [file 41598_2020_71365_MOESM1_ESM.docx]

**Supplementary Information**

**Electrochemical analysis of anionic analytes in weakly supported media using ‎electron transfer promotion effect: A case study on nitrite**

Alireza Khoshroo^a^, Ali Fattahi^a,b^*

*^a^ Pharmaceutical Sciences Research Center, Health Technology Institute, Kermanshah University of Medical Sciences, Kermanshah, Iran*

*^b^ Medical Biology Research Center, Health Institute, Kermanshah University of Medical Sciences, Kermanshah, Iran*

*E-Mail:* [*a.fatahi.a@gmail.com*](mailto:a.fatahi.a@gmail.com)

**1. Fabrication of ePAD**


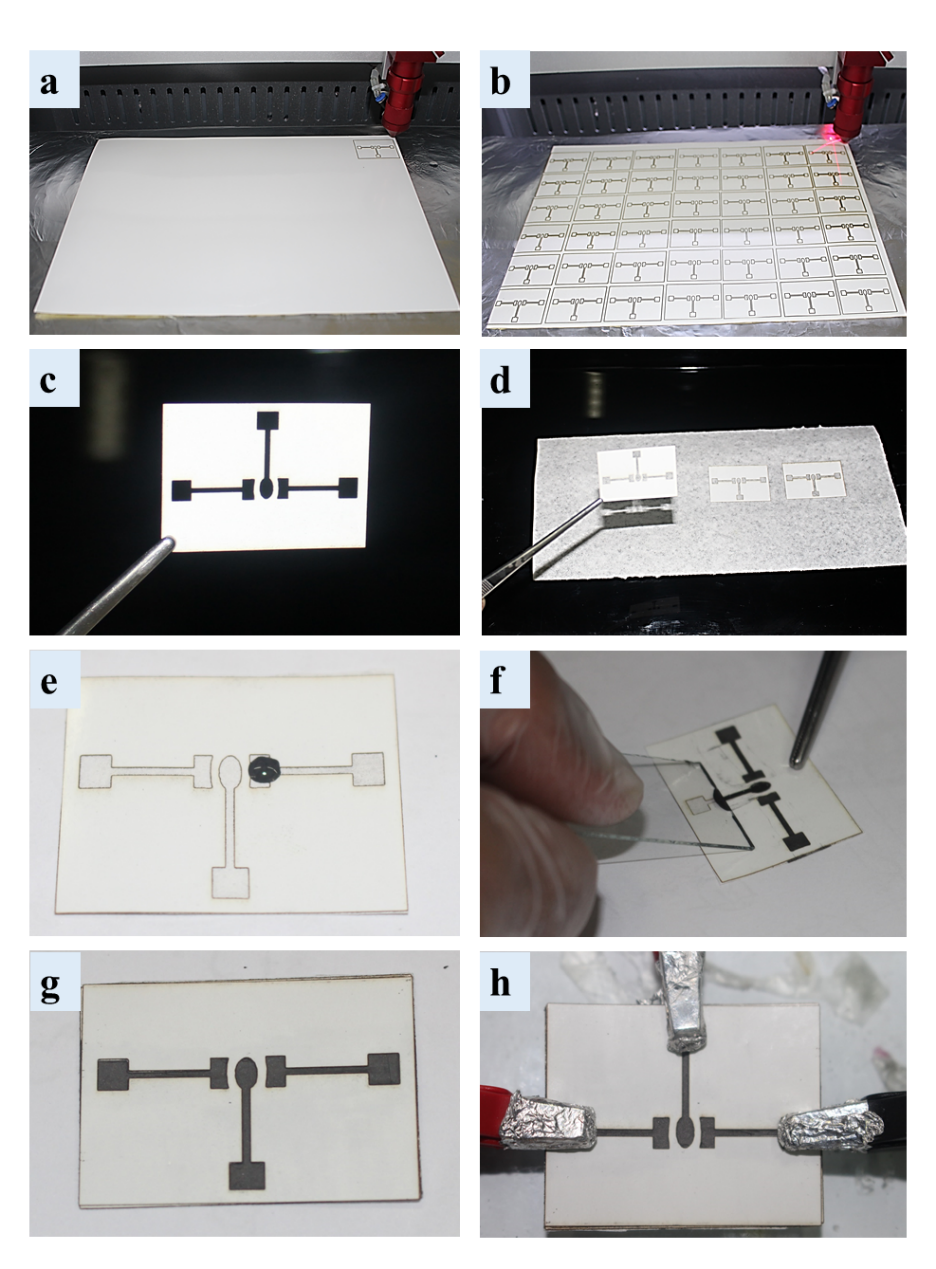


**Fig. S1.** (a-f) Different steps of the ePADs preparation.

Table S1. The Effect of various speed of the laser cutter on the reproducibility of pattern. The mean surface area was calculated for the ten patterns.

| speed | surface area (cm^2^) | RSD % |
| --- | --- | --- |
| 5 | 1.38 | 5.1 |
| 7.5 | 1.32 | 4.5 |
| 10 | 1.23 | 3.9 |
| 12.5 | 1.29 | 6.5 |
| 15 | 1.52 | 7.8 |

Table S2. The Effect of power of the laser cutter on the reproducibility of patterns. The mean surface area was calculated for the ten patterns.

| power% | surface area (cm^2^) | RSD % |
| --- | --- | --- |
| 10 | 1.15 | 4.3 |
| 12.5 | 1.23 | 3.9 |
| 15 | 1.47 | 6.2 |
| 17.5 | 1.61 | 7.6 |

**3. Characterizations of ePAD**

**Fig. S2.** SEM image of ePAD (A) and Au/ePAD (B).

**Fig. S3. (a)** XRD patterns and (b) Energy-dispersive X-ray spectrum of Au/ePAD.

**Fig. S4.** The peak currents for ePADs constructed by different ink ‎**in** different Days. Five ePAD constructed by the same ink every day, and three replicate measurements for each ePAD were recorded.

**Fig. S5.** Optimization of: (A) pH of nitrite solution (B) incubation time of NTX, (C) pH of NTX solution. DPV responses were performed in 0.1 mM nitrite.


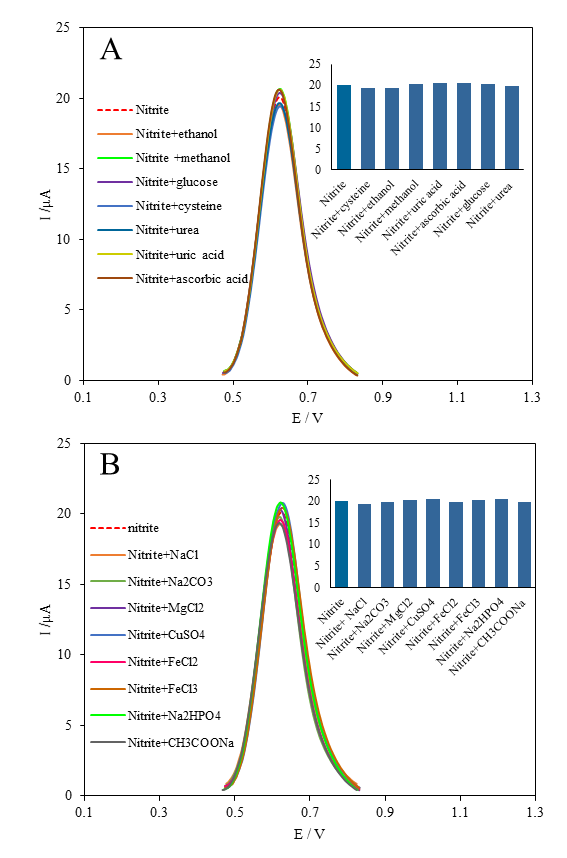


Fig. S6. DPV of NTX−Au/ePAD for the mixture of 0.1 mM nitrite and (A) 10-fold concentration of cysteine, ethanol, methanol, uric acid, ascorbic acid, glucose, urea and (B) 20-fold concentration of NaCl, Na_2_CO_3_, MgCl_2_, CuSO_4_, FeCl_2_, FeCl_3_, Na_2_HPO_4_, CH_3_COONa. DPV parameters: amplitude, 0.05 V; pulse width, 0.2 s; sampling width, 0.05 s; pulse period, 0.5 s.

**Table S3.** A comparison of the performance of several sensors for the detection of nitrite.

| Electrode materials | Linear range (μM) | Detection limit (μM) | Ref. |
| --- | --- | --- | --- |
| Ag nanoplates | 10-1000 | 1.2 | ^1^ |
| Multi-walled carbon nanotubes/gold nanoparticles | 0.05-250 | 0.01 | ^2^ |
| Fe_2_O_3_ NPs decorated reduced graphene oxide | 0.05–780 | 0.015 | ^3^ |
| CuO with H-C_3_N_4_ and reduced graphene oxide | 0.2-110 | 0.093 | ^4^ |
| Au nanoparticles on choline chloride | 0.4-750 | 0.1 | ^5^ |
| Tunnel structured MnO_2_ nanoparticle | 10-800 | 0.5 | ^6^ |
| Rose-like Au NPs/MoS_2_ nanoflower/graphene | 5-5000 | 1.0 | ^7^ |
| Silver/halloysite nanotube/ molybdenum disulfide nanocomposite | 2-245 | 0.7 | ^8^ |
| Ferrocene derivatives | 1.0-50 | 0.3 | ^9^ |
| Graphene nanosheets and gold nanoparticles | 0.3-720 | 0.1 | ^10^ |
| NTX−Au | 0.05–1400 | 0.002 | This work |

**Table S4** Determination of nitrite in real water samples.

| Sample | Spiked (μM) | Found (μM) | | t_exp_ ^a^ |
| --- | --- | --- | --- | --- |
|  |  | ePAD, (n=3) | Griess method, (n=3) |  |
| Deionized water | 0 | ND^b^ | ND | - |
|  | 10 | 9.78 ± 0.05 | 9.84 ± 0.06 | 1.4 |
| Tap water | 0 | 4.63 ± 0.07 | 4.73 ± 0.1 | 1.0 |
|  | 10 | 14.51 ± 0.14 | 14.63 ± 0.12 | 0.9 |
| Mineral water | 0 | 0.9 ± 0.03 | 1.0 ± 0.05 | 1.7 |
|  | 10 | 10.9 ± 0.15 | 11.1 ± 0.12 | 1.3 |
| ^a^ t_exp_ shows the experimental student-t values, (t _4, 0.05_ = 2.77)  ^b^ Not detected. | | | | |

**References**

1. Wang, Z., Liao, F., Guo, T., Yang, S. & Zeng, C. Synthesis of crystalline silver nanoplates and their application for detection of nitrite in foods. *J. Electroanal. Chem.* **664**, 135–138 (2012).

2. Afkhami, A., Soltani-Felehgari, F., Madrakian, T. & Ghaedi, H. Surface decoration of multi-walled carbon nanotubes modified carbon paste electrode with gold nanoparticles for electro-oxidation and sensitive determination of nitrite. *Biosens. Bioelectron.* **51**, 379–385 (2014).

3. Radhakrishnan, S., Krishnamoorthy, K., Sekar, C., Wilson, J. & Kim, S. J. A highly sensitive electrochemical sensor for nitrite detection based on Fe2O3 nanoparticles decorated reduced graphene oxide nanosheets. *Appl. Catal. B Environ.* **148**–**149**, 22–28 (2014).

4. Li, Y. *et al.* A novel electrochemical sensor based on CuO/H-C3N4/rGO nanocomposite for efficient electrochemical sensing nitrite. *J. Alloys Compd.* **798**, 764–772 (2019).

5. Wang, P., Mai, Z., Dai, Z., Li, Y. & Zou, X. Construction of Au nanoparticles on choline chloride modified glassy carbon electrode for sensitive detection of nitrite. *Biosens. Bioelectron.* **24**, 3242–3247 (2009).

6. Dai, Y., Huang, J., Zhang, H. & Liu, C. C. Highly sensitive electrochemical analysis of tunnel structured MnO2 nanoparticle-based sensors on the oxidation of nitrite. *Sensors Actuators B Chem.* **281**, 746–750 (2019).

7. Han, Y., Zhang, R., Dong, C., Cheng, F. & Guo, Y. Sensitive electrochemical sensor for nitrite ions based on rose-like AuNPs/MoS2/graphene composite. *Biosens. Bioelectron.* **142**, 111529 (2019).

8. Ghanei-Motlagh, M. & Taher, M. A. A novel electrochemical sensor based on silver/halloysite nanotube/molybdenum disulfide nanocomposite for efficient nitrite sensing. *Biosens. Bioelectron.* **109**, 279–285 (2018).

9. Feng, X.-Z. *et al.* A Facile Electrochemical Sensor Labeled by Ferrocenoyl Cysteine Conjugate for the Detection of Nitrite in Pickle Juice. *Sensors* **19**, 268 (2019).

10. Wang, P. *et al.* Development of a paper-based, inexpensive, and disposable electrochemical sensing platform for nitrite detection. *Electrochem. commun.* **81**, 74–78 (2017).
